# Deep Learning Assisted Automated Assessment of Thalassaemia from Haemoglobin Electrophoresis Images

**DOI:** 10.3390/diagnostics12102405

**Published:** 2022-10-03

**Authors:** Muhammad Salman Khan, Azmat Ullah, Kaleem Nawaz Khan, Huma Riaz, Yasar Mehmood Yousafzai, Tawsifur Rahman, Muhammad E. H. Chowdhury, Saad Bin Abul Kashem

**Affiliations:** 1 Department of Electrical Engineering, College of Engineering, Qatar University, Doha P.O. BOX 2713, Qatar; 2 Artificial Intelligence in Healthcare, Intelligent Information Processing Lab, National Center of Artificial Intelligence, University of Engineering and Technology Peshawar, Peshawar 25000, Pakistan; 3 Department of Biomedical Sciences and Engineering, Koc University Istanbul, Istanbul 34450, Turkey; 4 Department of Computer Science, University of Engineering and Technology Mardan, Mardan 23200, Pakistan; 5 Department of Pathology and Laboratory Medicine, Lady Reading Hospital, Peshawar 25000, Pakistan; 6 Department of Pathology and Blood Bank, Hayatabad Medical Complex/Khyber Girls Medical College, Peshawar 25000, Pakistan; 7 Institute of Pathology and Diagnostic Medicine, Khyber Medical University, Peshawar 25100, Pakistan; 8 Department of Computer Science, AFG College with the University of Aberdeen, Doha P.O. BOX 33199, Qatar

**Keywords:** automated lane extraction, convolutional neural network, object detection, haemoglobin electrophoresis

## Abstract

Haemoglobin (Hb) electrophoresis is a method of blood testing used to detect thalassaemia. However, the interpretation of the result of the electrophoresis test itself is a complex task. Expert haematologists, specifically in developing countries, are relatively few in number and are usually overburdened. To assist them with their workload, in this paper we present a novel method for the automated assessment of thalassaemia using Hb electrophoresis images. Moreover, in this study we compile a large Hb electrophoresis image dataset, consisting of 103 strips containing 524 electrophoresis images with a clear consensus on the quality of electrophoresis obtained from 824 subjects. The proposed methodology is split into two parts: (1) single-patient electrophoresis image segmentation by means of the lane extraction technique, and (2) binary classification (normal or abnormal) of the electrophoresis images using state-of-the-art deep convolutional neural networks (CNNs) and using the concept of transfer learning. Image processing techniques including filtering and morphological operations are applied for object detection and lane extraction to automatically separate the lanes and classify them using CNN models. Seven different CNN models (ResNet18, ResNet50, ResNet101, InceptionV3, DenseNet201, SqueezeNet and MobileNetV2) were investigated in this study. InceptionV3 outperformed the other CNNs in detecting thalassaemia using Hb electrophoresis images. The accuracy, precision, recall, f1-score, and specificity in the detection of thalassaemia obtained with the InceptionV3 model were 95.8%, 95.84%, 95.8%, 95.8% and 95.8%, respectively. MobileNetV2 demonstrated an accuracy, precision, recall, f1-score, and specificity of 95.72%, 95.73%, 95.72%, 95.7% and 95.72% respectively. Its performance was comparable with the best performing model, InceptionV3. Since it is a very shallow network, MobileNetV2 also provides the least latency in processing a single-patient image and it can be suitably used for mobile applications. The proposed approach, which has shown very high classification accuracy, will assist in the rapid and robust detection of thalassaemia using Hb electrophoresis images.

## 1. Introduction

Haemoglobin, an essential component of red blood cells, carries oxygen from the lungs to various tissues of the body. The adult haemoglobin molecule (haemoglobin A or HbA) is composed of two alpha and two beta chains. A variant of haemoglobin, HbA2 (composed of two alpha and two delta chains), is present in minor proportions in the blood (less than 3.5% of total Hb in normal individuals). Genetic mutations in beta-globin genes result in reduced production or the lack of production of adult haemoglobin. Depending on the type and severity of mutations, symptoms vary from mild subclinical anaemia (beta thalassaemia trait) to severe life-threatening anaemia requiring repeated blood transfusions (beta thalassaemia major). In Pakistan, an estimated 80–90 thousand patients are registered in various thalassaemia treatment centers, managed by various public and private sector organizations [1]. It is important to screen for silent carriers of thalassaemia (termed beta thalassaemia trait) as the offspring of two silent carrier parents have a 25% chance of inheriting the severe transfusion-dependent form of thalassaemia [2]. The diagnosis of thalasssaemia requires careful evaluation of patient history and clinical examination, microscopic examination of the peripheral blood film, and the assessment of various haemoglobin variants using haemoglobin electrophoresis. Electrophoresis testing is a common practice for thalassaemia screening, involving the separation of molecules that have the capability to acquire electric charges from a nearby electric field. This process is conducted in the presence of suspended ions, called a buffer solution. In the buffer solution, ions migrate between two electrodes, i.e., they travel towards the oppositely charged electrodes. In a normal adult, HbA is the most dominant form of haemoglobin (at 96–98%), whereas HbA2 occurs at a level of about 2–3.5%. In beta thalassaemia trait, a compensatory increase in the HbA2 level is seen, which may rise up to 7% HbA2. In beta thalassaemia major, almost all of the red blood cells consist of the fetal variant of haemoglobin (HbF) [3]. The interpretation of Hb electrophoresis remains a challenge in most low- and middle-income countries (LMICs) due to a lack of standardized protocols and expertise. To tackle these issues, in the current approach to the interpretation of Hb electrophoresis testing, computer-aided detection based on Hb electrophoresis test images is required. The automated system should be able to accurately interpret the electrophoresis test images for the diagnosis of thalassaemia traits in patients.

Thalassaemia is the most common single-gene disorder globally. A high prevalence of thalassaemia and other haemoglobin disorders appears to be an evolutionary mechanism against malaria infection. The prevalence of thalassaemia is high in sub-Saharan Africa, the Mediterranean region, the Middle East, South East Asia and South Asia. However, with increased global migration, a higher number of cases are being seen in Europe and the Americas. At a genetic and molecular level, beta-globin chain production is controlled by beta chain genes located on each of the pair of chromosome 11. More than 200 mutations have been reported in beta-globin genes. Mutation in one of the two genes results in mild, often subclinical reductions in cell volume and haemoglobin levels. Such persons are labeled ‘carriers’ or ‘heterozygous’. Persons carrying two mutations are labeled ‘homozygous’. A mutation may severely reduce or completely block the production of beta chains (β+ or β0 thalassaemia, respectively). Consequently, based on the type of mutation, and whether both genes are affected, the disease’s clinical phenotype ranges from asymptomatic to mild, moderate and severe [4].

In patients with homozygous beta thalassaemia, symptoms begin to appear after the first trimester of life, when HbF (fetal) production ceases and is not replaced by normal HbA (adult). If untreated, infants develop severe anaemia, enlargement of the liver and spleen, and failure to thrive. With appropriate treatment, patients have been shown to survive until the fifth or sixth decades of life. However, in low–middle-income countries, the average life expectancy is still not above 20 years. Patients are dependent on blood transfusions for their entire lives. Repeated blood transfusions cause the accumulation of excessive iron, causing organ damage. Furthermore, transfusion-transmitted infections (TTIs), such as viral hepatitis B and C and human immunodeficiency virus (HIV), are quite common. The only long-term sustainable solution is the prevention of thalassaemia major through the identification of carriers of beta thalassaemia [4].

Thalasssaemia is a genetic disorder and is a chronic disease. The body of the thalassaemic patient makes an inadequate or an abnormal form of haemoglobin. This leads to a life-threatening form of anaemia and for survival the patients require blood transfusions on a regular basis [5]. The World Health Organization (WHO) has declared the control of haemoglobin disorder, particularly β-thalassaemia, in developing countries, as a national and international health priority [6]. In Pakistan, every year an estimated 5000–9000 children are born with β-thalassaemia and there are almost 9.8 million carriers at a national level [7]. However, its spread can be controlled through the early detection of thalassaemia carriers, i.e., by avoiding marriages between people who are carriers of thalassaemia traits [8]. In order to prevent β-thalassaemia, collective measures can be carried out such as carrier identification, prenatal diagnosis and, more importantly, genetic counseling. For the screening and assessment of thalassaemia patients, different techniques are discussed in the following sections.

The severity and high impact of thalassaemia on human life demands accurate and efficient solutions for thalassaemia diagnosis. Researchers and medical experts are equally involved in the process of experimentation to devise a computer-aided and automated solution for the detection of this deadly disease. Generally, for the detection of thalassaemia, complete blood count (CBC) and haemoglobin tests are performed [9]. There are numerous approaches and techniques [10,11], in which different CBC parameters, including white blood cells (WBC), red blood cells (RBC), haemoglobin (HB), haematocrit (HCT), mean corpuscular volume (MCV), mean corpuscular haemoglobin (MCH) and mean corpuscular haemoglobin concentration (MCHC), are used as features with machine learning classifiers, such K-nearest neighbor (KNN), multi-layer perceptron (MLP), decision tree support vector machine (SVM) and the latest deep learning paradigm, i.e., convolutional neural networks (CNNs), to devise an automated solution for thalassaemia detection.

For several decades, Hb electrophoresis has been used for the screening and assessment of thalassaemia in clinical practices [12]. Although high-performance liquid chromatography (HPLC) [13] and polymerase chain reaction (PCR) analysis can also be used for thalassaemia diagnosis [14], Hb electrophoresis is the cheapest and most commonly used method. In routine clinical diagnostics in developing countries, Hb electrophoresis is the most commonly used method. In more developed countries, it is being replaced by high-performance liquid chromatography (HPLC), and in some cases polymerase chain reaction (PCR) for thalassaemia-specific mutation analysis.

**Figure 1:**
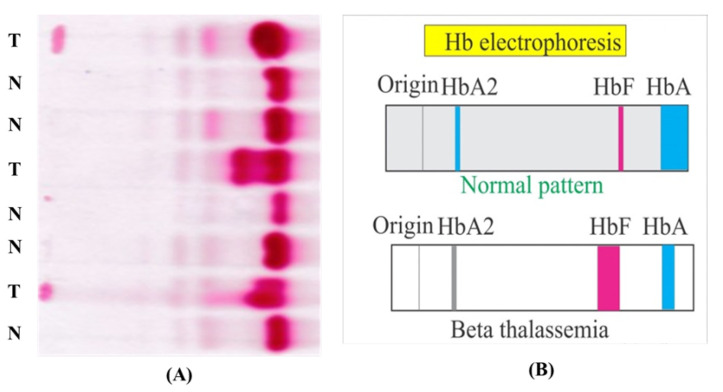
(**A**) Raw Hb electrophoresis images from eight subjects, and (**B**) standard normal and beta thalassaemia patterns in Hb electrophoresis images.

The test procedure of Hb electrophoresis is simple; however, multiple steps are involved. Briefly, red cells are lysed to liberate the haemoglobin into a solution. This lysate is then applied on a suitable electrophoresis medium (e.g., a cellulose acetate strip) to perform electrophoresis. Due to variations in ionic charge, the various Hb variants travel different distances. This strip is then dipped into a Ponceau staining solutionto highlight the bands. Hb electrophoresis test images contain the blood samples of more than one patient (up to eight patients) on a single cellulose sheet, as shown in Figure 1A,B. Figure 1A shows the Raw Hb electrophoresis slide images of eight subjects, where normal patients are denoted by N and thalassaemia patients are denoted by T, and Figure 1B shows standard normal and beta thalassaemia patterns of Hb electrophoresis images. In the figure, haemoglobin A (HbA), also known as adult haemoglobin, haemoglobin A1, is the most common human haemoglobin tetramer, accounting for over 97% of the total red blood cell haemoglobin. Haemoglobin is an oxygen-binding protein, found in erythrocytes, which transports oxygen from the lungs to the tissues. HbA2, composing two chains α and two δ chains, is a minor component of the haemoglobin present in normal adult red blood cells, accounting for about 2.5% of the total haemoglobin in healthy individuals. For thalassaemia patients, its content above 3.5%. Moreover, HbF is the predominant form of haemoglobin in red cells during fetal life. Just after birth, the level of HbF decreases gradually to <1%, and is replaced mainly by adult haemoglobin (HbA) (97%). To automate the process, each patient’s sample lane has to be extracted for further processing, which can be achieved using lane extraction techniques. In the following paragraph, recent works on lane extraction are discussed.

Ivan Bajla et al. [15] proposed a gel image analysis method for band detection, which is performed manually, i.e., users have to select or reject the extracted lane images manually. In [16], Helena et al. proposed a method for the removal of distortion from the gel images, which resolves the problem of non-uniformity in the process of electrophoresis via contrast adjustment and median filtering. They obtained the peaks based on the relative intensity. Abeykoon et al. [17] presented a lane extraction method on the basis of the size of the molecular weight and its distance traveled in the respective lane. Samira et al. [18] presented a lane extraction method on the basis of the size of the molecular weight and its distance traveled in the respective lane. In [19], another lane extraction method was presented, which estimates the average lane width of gel electrophoresis images via k-means clustering. Local maxima are calculated on a small portion of the image as potential lane centers then lanes are segmented using local minima. Yang et al. [20] performed lane extraction on gel electrophoresis images, in which the image was preprocessed by removing the grid texture from the background, then a watershed algorithm was applied to calculate the centerlines of the bands, and finally the shortest path algorithm was used to recover the shape. All these existing techniques perform well, with reasonable accuracy, but these techniques require input from the user to adjust the region of interest and other different parameters manually.

It is apparent from the above discussions that challenges exist with the current electrophoresis technique used for the detection of thalassaemia patients using computer-aided diagnostics systems with minimal human intervention. One is to automatically extract the slide image for a single patient and classify the slide with a reasonable degree of accuracy based on the electrophoresis image. However, thalassaemia diagnosis with electrophoresis images still requires human experts to interpret the images by comparing them with standard normal images. Several novel contributions are reported in this work:

- A relatively large electrophoresis image dataset was created from 824 patients (normal and thalassaemia);
- An automatic lane extraction technique has been proposed, which efficiently detects and extracts lanes from the given cellulose sheet and extract a single patient’s electrophoresis image;
- Seven different pre-trained CNN models were investigated for the classification of electrophoresis images; and
- Image visualization techniques have proposed to investigate where the CNN model is learning from.

The article is organized into the following sections: Section 1 discusses the motivation, background of novelty of this work. Section 2 discusses the background of popular CNN models and Section 3 describes the visualization techniques. Section 4 illustrates the methodology of the work. Section 5 illustrates the results and provides a discussion of the experiments carried out in this study, whereas Section 6 concludes the work.

## 2. Deep Convolutional Neural Networks

Deep CNNs have been popularly used in image classification due to their superior performance compared to other machine learning paradigms. The network structure automatically extracts the spatial and temporal features of an image. The approach of transfer learning has been successfully incorporated in many applications [21,22], especially where large datasets can be hard to find. Thus, this has created the opportunity to utilize smaller datasets and has also reduced the time required to develop a deep learning algorithm from scratch [23,24]. For COVID-19 detection, nine pre-trained deep learning CNNs, such as ResNet18, ResNet50, ResNet101 [25], DenseNet201 [26], and InceptionV3 [27,28], were pre-dominantly used in the literature. Residual networks (in short ResNets), which have several variants, solve vanishing gradient and degradation problems [25] and learn from residuals instead of features [29]. A dense convolutional network (in brief, DenseNet) requires a smaller number of parameters than a conventional CNN, as it does not learn redundant feature maps. DenseNet has layers with direct access to the original input image and loss function gradients. InceptionV3 is a CNN architecture from the inception family that makes several improvements, including using label smoothing, factorized 7 × 7 convolutions, and the use of an auxiliary classifier to propagate label information lower down the network (along with the use of batch normalization for layers in the side head). This network scales in ways that strive to use the added computation as effectively as possible through correctly factorized convolutions and aggressive regularization [28]. SqueezeNet involves three main strategies: (1) replacing 3 × 3 filters with 1 × 1 filters, (2) decreasing the number of input channels to 3 × 3 filters and (3) down-sampling the network so that convolution layers have large activation maps. Strategies 1 and 2 are based on judiciously decreasing the quantity of parameters in a CNN, while attempting to preserve accuracy. Strategy 3 entails maximizing accuracy on a reasonable number of parameters. In MobileNetV2, each block contains a 1 × 1 expansion layer, in addition to a depthwise and a pointwise convolutional layer. Unlike V1, the pointwise convolutional layer of V2, known as the projection layer, projects data with a high number of channels into a tensor with a much lower number of channels. The bottleneck residual block has the output of each block as a bottleneck. A 1 × 1 expansion convolutional layer will expand the number of channels depending on the expansion factor in the data before they undergo the depthwise convolution process. The second novel component in MobileNetV2 is the residual connection. The residual connection exists to help the flow of gradients through the network. Each layer of MobileNetV2 involves batch normalization and a rectilinear unit (ReLU) as the activation function. However, the output of the projection layer does not have an activation function. The full MobileNetV2 architecture consists of 17 bottleneck residual blocks in a row, followed by a regular 1 × 1 convolution, a global average pooling layer. In this work, seven different pre-trained CNN models (three variants of ResNet, InceptionV3, DenseNet201, SqueezeNet and MobileNetV2) were investigated.

## 3. Visualization Techniques

**Figure 2:**
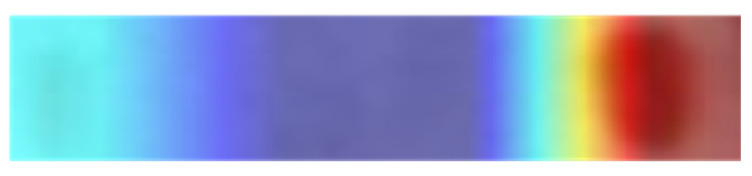
Score-CAM heat map on Hb electrophoresis test image.

Increasing interest in the internal mechanisms of CNNs and the reasoning behind the specific decisions made by networks has led to the development of visualization techniques. The visualization techniques help in obtaining a better visual representation in order to interpret the decision-making processes of CNNs. These also increase the model’s transparency by visualizing the logic behind the inference that can be interpreted in a way that is easily understandable to humans, thereby increasing confidence in the outcomes of neural networks. Amongst the various visualization techniques, such as SmoothGrad [30], Grad-CAM [31], Grad-CAM++ [32] and Score-CAM [33], the recently proposed Score-CAM was used in this work due to its promising performance. Score-CAM avoids the dependence on gradients by obtaining the weight of each activation map through its forward passing score on the target class.The final result is obtained through a linear combination of weights and activation maps. A sample image visualization obtained using Score-CAM is shown in Figure 2, where the heat map indicates regions that dominantly contributed in the decision-making of the CNN. This can be helpful in understanding how the network is making its decision and also in improving the confidence of the end-user when it can be confirmed that at all times the network is making decisions using the Hb electrophoresis images. In regard to specific lane images from the dataset, it is clearly visiblethat the model was making a decision based on the HbF and HbA bands, as this part is reddish. The blue zone part means this part made less of a contribution to the decision made by the network.

## 4. Methodology

**Figure 3:**
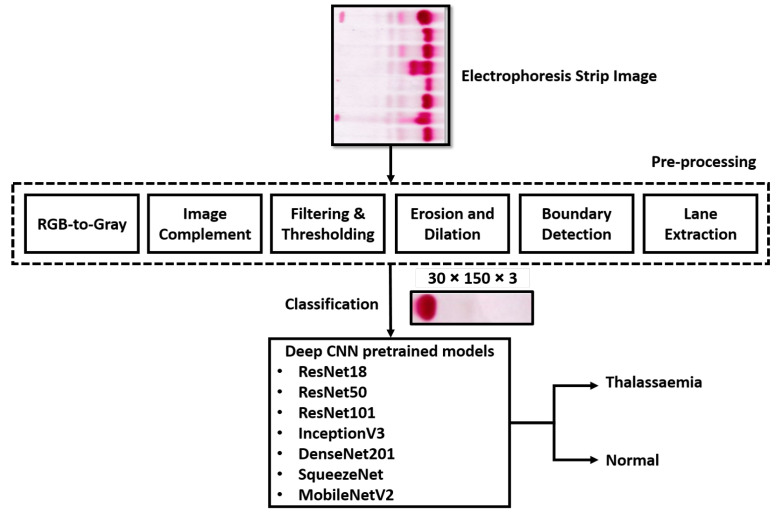
Overview of the complete methodology used in this study.

In this section, a deep learning method for the automated assessment of thalassaemia using electrophoresis images is presented. The proposed methodology includes database development, pre-processing of electrophoresis images, object detection, lane extraction and finally the classification of normal and thalassaemia classes. The steps of the proposed methodology are shown in Figure 3, and are detailed in the following subsections.

### 4.1. Database Development

**Figure 4:**
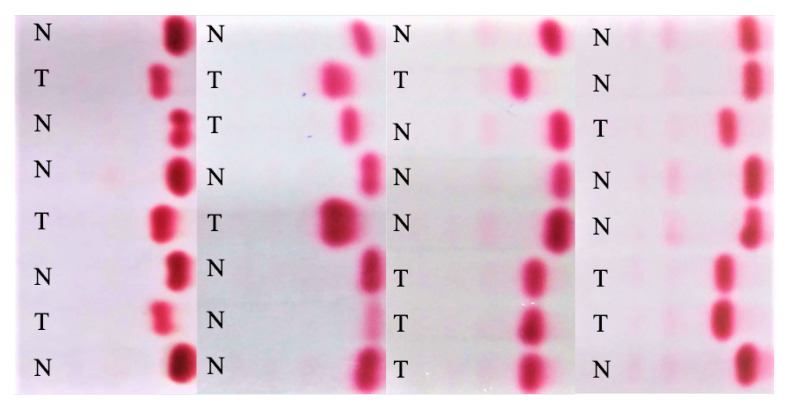
Sample electrophoresis raw images from the dataset.

**Figure 5:**
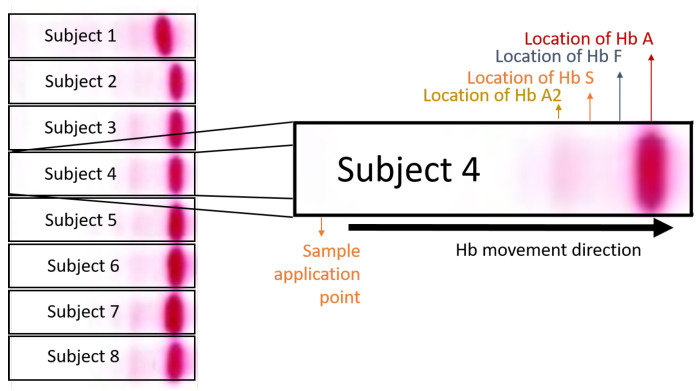
Description of a single electrophoresis image from a patient in an eight-patient strip image.

The dataset used in this research was recorded over the course of two years at Lady Reading Hospital in Peshawar, Pakistan. The strips were scanned and anonymized. The assessment and labeling of Hb electrophoresis images were performed by three independent haematologists. These were selected based on their post-graduate qualifications as haematologists. All three were responsible for diagnostic clinical work in their respective hospitals. Figure 4 shows sample raw electrophoresis images from the dataset. The database consisted of 103 strips containing electrophoresis images from 824 subjects. Each strip contained 8 patients’ images. A total of 824 images were analyzed. Images with a clear consensus on the quality of electrophoresis were included. Cases with poor images or diagnoses other than beta thalassaemia trait, as well as some normal images causing a data imbalance, were excluded. Finally, a total of 524 images were included in the analysis. A selection process was performed in order to have 50% in each class (thalassaemia or normal). After some preparations, each subject’s sample was applied on the test strip at the point of application, as shown in Figure 5. The bands were formed by Hb variants according to their weights. HbA moves faster and makes the first band, due to its lighter weight. Similarly, HbA2 moves slowly and is left behind, thus making the last band in the image due to its heavier weight. After the separation of haemoglobin, the diagnosis can be made on the basis of the percentage of each variant in the image.

### 4.2. Pre-Processing

**Figure 6:**
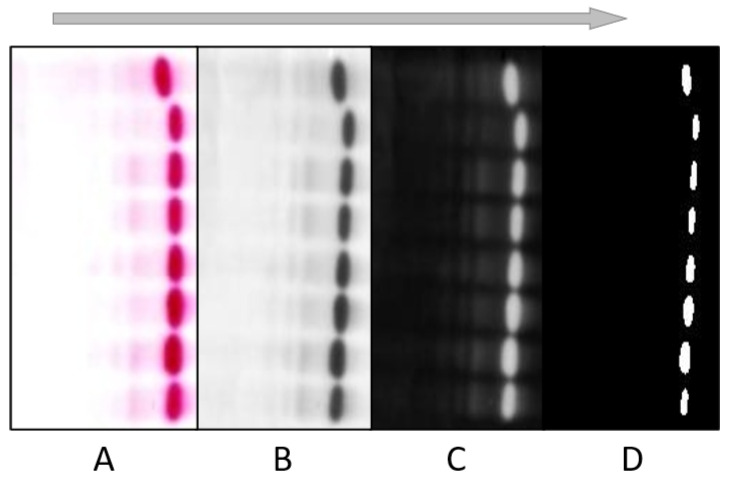
Pre-processing techniques applied to a sample test image: (**A**) raw (**B**) RGB to grayscale conversion, (**C**) image complementation, (**D**) thresholding of electrophoresis images.

Several pre-processing steps were applied to the raw images to extract the slides for each patient. The most common steps were (i) filtering and thresholding, (ii) object detection, (iii) erosion and dilation and (iv) boundary detection and lane extraction. Figure 6 shows different filtering and thresholding steps, depicting raw images (A), RGB to grayscale conversion (B), complementing the image (C) and image thresholding (D). Each step is separately discussed in the following subsections.

#### 4.2.1. Filtering and Thresholding

In electrophoresis images, most of the information lies in the low frequencies, whereas noise is present in high-frequency pixel values. A Gaussian filter, also known as blur filter, with a kernel size of 5 × 5 was applied to the complimented images. Gaussian filters are good for noise removal but the filtered image becomes blurred [34]. The noise removal capability and the associated blurring of the image can be adjusted via the selection of the appropriate parameters.

Thresholding is commonly used for the purpose of segmentation. In [35] a gradient analysis approach for the calculation of the threshold value was adopted. Otsu thresholding was used in this work to minimize the inter-class variance among the background and foreground. Afterwards, the optimum value of threshold was used for the conversion of grayscale images into binary images.

#### 4.2.2. Object Detection

There were data from eight subjects in every scanned image of a strip. To separate each of them from one another, an object detection technique was used. This process involves two further steps, including erosion- dilation and boundary detection to better separate the lanes.

#### 4.2.3. Erosion and Dilation

Erosion and dilation are two fundamental nonlinear operations related to the shape of the features in an image [36]. Dilation adds pixels to the boundary of the object in an image, whereas erosion removes pixels on the object boundaries. In the proposed method, an erosion operator was employed twice, using two different scales to erode the image. This was carried out because some of the blood molecules in consecutive lanes combined with one another and it became difficult to detect them separately. In order to address this problem, we suggested first applying the erosion operator, followed by dilation to compensate for the erosion. For this process, a structuring element of 5 × 5 was selected.

#### 4.2.4. Boundary Detection

**Figure 7:**
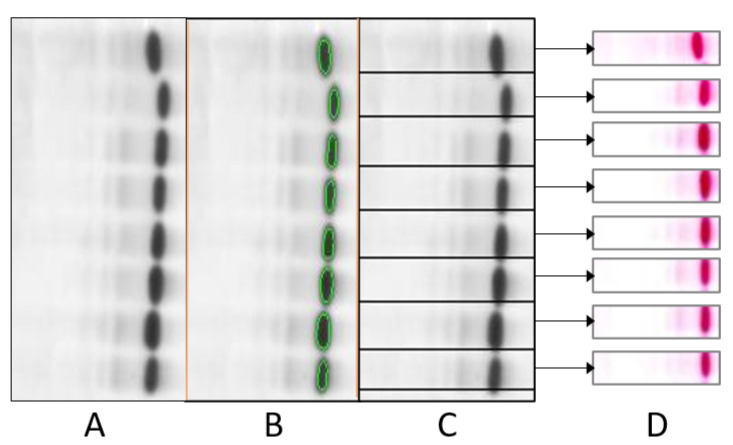
Pre-processing techniques of grayscale images for lane extraction: (**A**) grayscale image, (**B**) connected object detection, (**C**) extracted lane images and (**D**) cropped images.

In the proposed methodology, boundary detection was performed using connected components. The main function of connected-component analysis is in distinguishing large-sized connected foreground regions of an image from the background regions of the image. The connected pixels were clustered together, as illustrated in Figure 7C. Subsequently, based on the object boundaries, the point of separation for each lane was predicted. The algorithm was used with an 8 pixel connectivity value.

#### 4.2.5. Lane Extraction

For further analysis of electrophoresis test images, each lane had to be extracted from the image. For lane extraction, the first step was to determine the boundary pixels using boundary points obtained from the connected-component analysis, as shown in Figure 7B, and the next step was to crop the original RGB images using the obtained boundary pixels, as depicted in Figure 7C. As a result, the test image was divided into separated lanes, as shown in Figure 7D, where each lane represented the test result of an individual patient. The cropped images were converted into a fix-sized format. The chosen size was a 30 × 150 pixel RGB image (3-channels). This fixed sized images with cropped lanes were fed as inputs into the classifier.

### 4.3. Thalassaemia Detection

Seven different CNN models were trained, validated and tested separately using thalassaemia images for the classification of thalassaemia and non-thalassaemia (normal) results. The complete set of images was divided into 80% training and 20% testing subsets for five-fold cross validation and 20% of the training data were used for validation. Table 1 shows the number of training, validation and test images used for the experiments. To make the training image sets for both classes, the training images were augmented 14 times to make the number of images 2835 per fold. Three augmentation strategies (rotation, scaling and translation) were utilized to balance the training images. The rotation was performed from $3^{\circ }$ to $5^{\circ }$ clockwise. The scaling operation involves the magnification or reduction of the frame size of the image; 2.5% to 10% image magnifications were used in this work. Image translation was accomplished by translating images horizontally and vertically by 5% to 10%.

All the CNNs were implemented using the PyTorch library with Python 3.7 on an Intel Xeon CPU E5-2697 v4 @ 2.30 GHz and 64 GB RAM, with a 16 GB NVIDIA GeForce GTX 1080 GPU. Three comparatively shallow networks (MobileNetv2, SqueezeNet and ResNet18) and four deep networks (InceptionV3, ResNet 50, ResNet101 and DenseNet201) were evaluated in this study to investigate whether shallow or deep networks were more suitable for this application. Three different variants of ResNet were used to compare specifically the impact of shallow and deep networks with similar structures. The pre-trained CNN models were trained using the same training parameters and stopping criteria, as shown in Table 2. The stopping criteria meant that the training would be stopped if the validation losses did not decrease for five consecutive epochs, and epoch patience was used to decrease the learning rate if the validation loss did not decrease for a few consecutive epochs. Fifteen back-propagation epochs were used for the classification problem. Five-fold cross-validation results were averaged to produce the final receiver operating characteristic (ROC) curve, confusion matrix and evaluation matrices. The use of image augmentation and having a validation image set helped in avoiding the overfitting of the models [37].

(1) $$Accuracy=\frac{TP+TN}{TP+FN+FP+TN}\;$$

(2) $$Recall=\frac{TP}{TP+FN}\;$$

(3) $$Specificity=\frac{TN}{TN+FP}\;$$

(4) $$Precision=\frac{TP}{TP+FP}\;$$

(5) $$F1Score=\frac{2\times TP}{2\times TP+FP+FN}\;$$

### 4.4. Performance Matrix

The performance of different CNNs on the testing dataset was evaluated after the completion of the training and validation phases and was compared using six performance metrics: accuracy, recall, specificity, precision, area under the curve (AUC) and F1 scores. The matrices were calculated using Equations (1)–(5) with 95% confidence intervals (CIs). Accordingly, the CI for each evaluation metric was computed, as shown in Equation (6).

(6) $$Confidence\;intervals:\;r=z\times \sqrt{\frac{metric\times (100-metric)}{100^{2}\times N}}\;\;$$

where *N* is the number of test samples and *z* is the level of significance, that is, 1.96 for 95%.

Here, true positive (TP), true negative (TN), false positive (FP) and false negative (FN) values were used to denote the number of thalassaemia images identified as thalassaemia, the number of normal images identified as normal, the number of normal images incorrectly identified as thalassaemia images and the number of thalassaemia images incorrectly identified as normal images, respectively. In addition, the networks could be compared in terms of the processing time for each test image. The processing time here refers to the time elapsed per image by a network when processing the images as a batch with parallelization. The processing time is represented in Equation (7), where $t_{1}$ and $t_{2}$ are the start and end time for a network to classify an image I, respectively. All time is measured in seconds. Based on the results, it is evident that the method can work on a low-end computer, even if there is no GPU, on an embedded computer or on mobile technology in negligible time compared to the time of electrophoresis.

(7) $$\delta te=t_{2}-t_{1}\;$$

## 5. Results and Discussion

Seven different models were trained, validated and tested for the classification of thalassaemia and normal (non-thalassaemia) cases. The comparative performance of different CNNs in two-class classification between thalassaemia and normal images is shown in Table 3. It is apparent from Table 3 that all the evaluated pre-trained models performed very well in classifying thalassaemia and normal images in the two-class problem. Among the networks trained with thalassaemia and non-thalassaemia normal images, Inceptiov3 and MobileNetV2 performed equally well in classifying the images. We did not necessary find that deeper networks would perform better; rather, MobileNetV2 is a very good example of transfer learning and it outperformed other networks in this task. ResNet18 showed comparatively better performance than ResNet50 and 101, which are much deeper networks than ResNet18. It can be seen that apart from SqueezeNet, most of the shallow networks performed well in this task. This could be because electrophoresis-image-based classification was a comparatively straightforward problem for these pre-trained CNN models. However, the AUCs for InceptionV3, Densnet201 and Resnet18 were close to each other. However, that of InceptionV3 was slightly better than others, as shown in Table 3.

**Figure 8:**
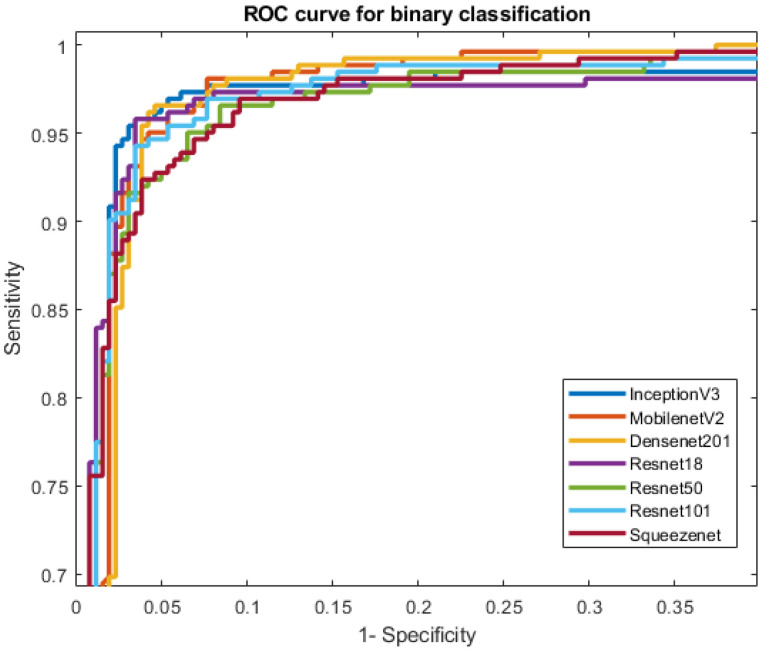
Comparison of the ROC curves for normal and thalassaemia classification using CNN models.

**Figure 9:**
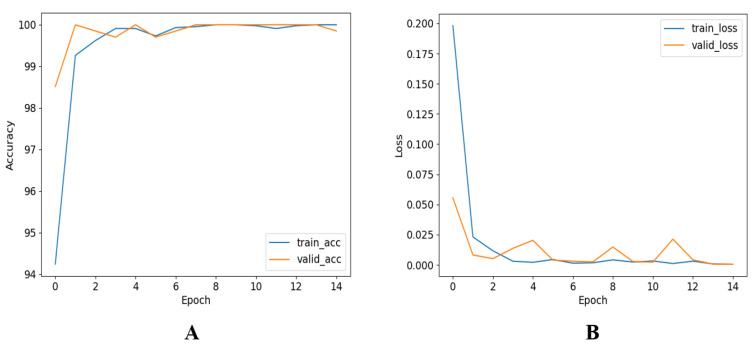
Training and validation (**A**) accuracy versus epoch and (**B**) losses versus epoch for the the best-performing trained CNN networks.

Figure 8 clearly shows that the ROC curves for InceptionV3 were the best among the seven different CNN models. However, it is obvious that most of the tested models performed very well in distinguishing thalassaemia and normal images.This reflects the fact that shallow or deep CNNs can distinguish between thalassaemia and normal images with very high reliability. Although all the CNNs exhibited better performance in classification, InceptionV3 showed the most outstanding performance in classifying thalassaemia and normal images. Even though, performance-wise, InceptionV3 was the best performer, it was comparatively slow in processing the test images, being the deepest network in the group. Figure 9 shows the training and validation accuracy versus epochs and loss versus epochs for the best-performing network (InceptionV3). It can also be seen that the network reached the lowest loss value relatively early.

**Figure 10:**
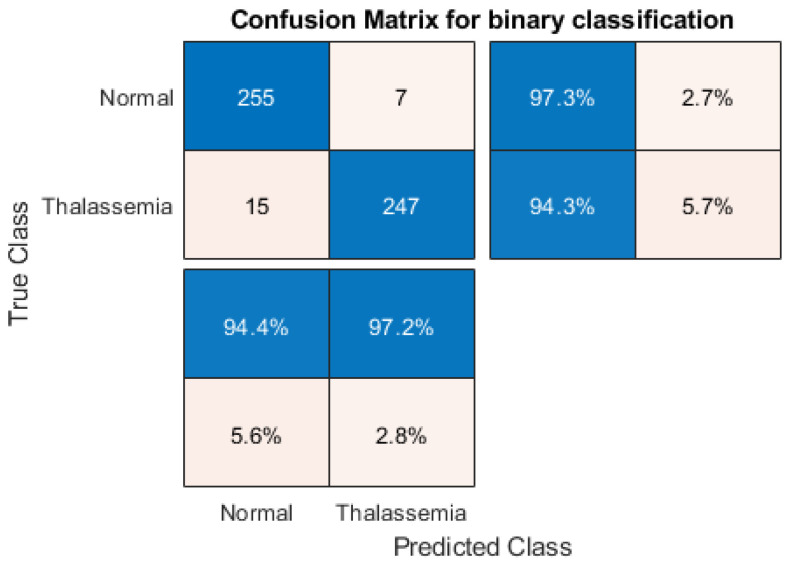
Confusion matrix for normal and thalassaemia classification for the best-performing model (InceptionV3).

**Figure 11:**
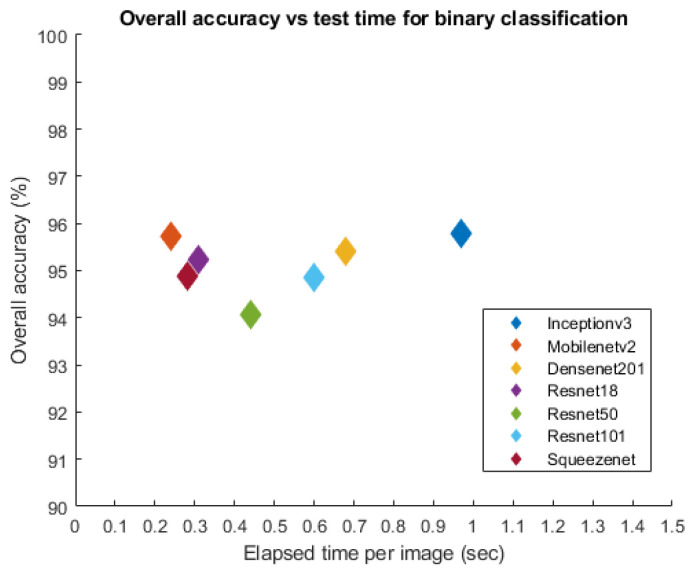
Comparison of accuracy versus the elapsed time per image using seven different networks for thalassaemia classification.

In summary, InceptionV3 demonstrated the highest classification accuracy of 95.8% in the classification of non-thalassaemia and thalassaemia images. Figure 10 shows the confusion matrix for the high-performing InceptionV3 model with thalassaemia and normal images. It can be noted that for the best-performing network, 15 out of 262 thalassaemia images were misclassified as normal images and 7 out of 262 normal images were misclassified as thalassaemia images. This is clearly an outstanding performance for any computer-aided classifier and this can significantly help in the fast screening of thalassaemia by clinicians immediately after acquiring the images. It can be seen that MobileNetV2 was the best-performing network according to elapsed time per image (δte), as shown in Figure 11, and it had the lowest number of parameters, as shown in Table 4. Finally, it can be observed that MobileNetV2, being a shallow network, produced very good accuracy, precision and recall values of 95.72%, 95.73% and 95.72%, respectively, which were very close to the accuracy, precision and recall of 95.8%, 95.84%, and 95.8%, respectively, for InceptionV3, which is a deep network.

**Figure 12:**
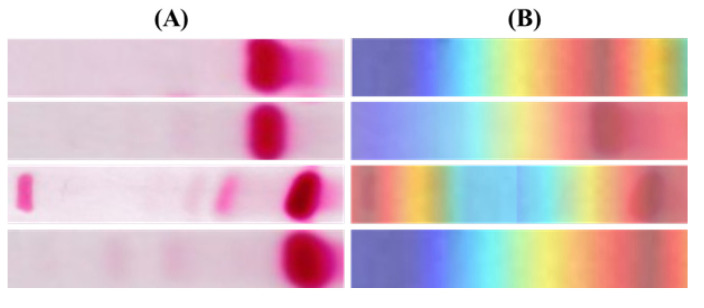
Score-CAM visualization of (**A**) Hb electrophoresis images, and (**B**) corresponding Score-CAM heat-map for different subjects.

In order to visualize the decision-making processes of the CNNs, visualization methods can be used to obtain a better visual representation. These also improve the transparency of the model by visualizing the reasoning behind the inference, enabling it to be interpreted in a way that can be easily understood by humans, and thereby increasing trust in the results of the CNNs. In this study, Score-CAM was used due to its promising performance in the previous works of the authors. Figure 12 provides some examples of Score-CAM visualizations, highlighting the regions used by the CNNs in making decisions. This visualization can help to increasing the level of confidence in the reliability of CNN models by confirming that the decision-making was based on relevant regions of the images. Figure 12A shows the raw Hb electrophoresis images and Figure 12B shows the Score-CAM heat-maps for those images, obtained using the best-performing CNN model. It is clearly visible that models made their decisions based on the HbF, HbA and HbA2 bands in the Hb electrophoresis images. It is worth noting here that the blue color in the Score-CAM images represents a lower contribution, whereas the red color represents a higher contribution.

The state-of-the-art performance of our proposed method was compared with the recently published works in the same problem domain. Table 5 summarizes a comparison of the results presented in this paper with those of others in the detection of thalassaemia using Hb electrophoresis images. In a few recent studies [10,38,39], detection accuracies of 93.7% were reported, using databases consisting of a small number of images. However, in our study, we used larger datasets than others and obtained consistent results. We also used lane extraction techniques and evaluated classification performance using seven different CNN models, which makes our method more robust and versatile, with 95.8% accuracy. Moreover, the performance of our model was justified using the Score-CAM based visualization technique to emphasize the importance of learning on the basis of the dominant regions identified by the CNN-based classification tasks.

Despite the promising results obtained in this study, there are few limitations of the proposed approach which require further investigation and can be considered as an extension of this study. A few of them are described as follows:

- The presence and concentration of HbA2 and HBF determines the severity of the disease. This classifier only classifies beta thalassaemia minor, which involves a specific concentration of Hb. In Southeast Asia, this affects about 15% of cases. These individuals normally do not have any clinically significant issues as long as they maintain Hb levels between 9 and 12 g/dL. There is no need for therapy. Moreover, since this is the first computer-aided diagnosis technique for electrophoresis images, this study is limited to binary classification.
- The populations may not be the same between different studies (e.g., different countries with different levels of healthcare). High confidence intervals were obtained, and the results for the proposed approach were the best among nine other methods, and thus there was probably a slight over-evaluation. The ground truth used was an expert evaluation, and this may not have been the case for the other methods. The fact that the processing of Hb electrophoresis images with deep learning was the best among other techniques has to be confirmed through a blind study designed for this purpose, in which the techniques are applied to the same patients.

## 6. Conclusions

In this study, a novel deep-learning-based approach to the screening of thalassaemia has been investigated for the automated assessment of thalassaemia. The primary task was to automatically extract the lanes from strips of electrophoresis images and to identify human subjects as being normal or abnormal in relation to thelassaemia. The proposed method was tested on data from 524 patients, showing a thalassaemia classification accuracy of 95.8% when using the InceptionV3 network. This was the first attempt that has been made to classify electrophoresis test images using a deep learning classification model, and the method achieved reasonable accuracy. The proposed technique avoids manual assessment and incorporates automated assessment and detection in the thalassaemia screening pipeline. The results also showed that through the use of electrophoresis images with CNNs, researchers can achieve good results compared to other expensive tests and techniques that can be adopted for thalassaemia screening. In the future, we intend to increase the number of electrophoresis image data in the developed database. Moreover, we intend to develop a hardware solution for automated thalassaemia screening.

## Figures and Tables

**Table 1:** Details of training, validation and test sets for classification problems.

Types	Total No. of Hb Electrophoresis Images/Class	Training without and with Image Augmentation		
		Training Set/ Fold	Validation Set/Fold	Test Set/ Fold
Normal	262	189 * 15 = 2835	21	52
Thalassaemia	262	189 * 15 = 2835	21	52

**Table 2:** Details of training parameters used for classification.

Training Parameters	
batch size	16
learning rate	0.001
epochs	15
epoch patience	4
stopping criteria	5
optimizer	Adam

**Table 3:** Comparison of the performance of different CNN models in thalassaemia classification.

Network	Accuracy (%)	Precision (%)	Recall (%)	F1-Score (%)	Specificity (%)	Elapsed Time for Testing (perImage) (δte) (Seconds)	Area under the Curve (AUC)
InceptionV3	95.80 ± 1.72	95.84 ± 2.42	95.80 ± 2.43	95.80 ± 2.43	95.80 ± 2.43	0.97	96.88
MobileNetV2	95.61 ± 1.75	95.66 ± 2.47	95.60 ± 2.48	95.63 ± 2.48	95.60 ± 2.48	0.24	95.60
Densnet201	95.42 ± 1.79	95.44 ± 2.53	95.42 ± 2.53	95.42 ± 2.53	95.42 ± 2.53	0.68	96.75
ResNet18	95.23 ± 1.82	95.26 ± 2.57	95.22 ± 2.58	95.22 ± 2.58	95.22 ± 2.58	0.31	96.65
ResNet50	94.08 ± 2.02	94.12 ± 2.85	94.08 ± 2.86	94.08 ± 2.86	94.08 ± 2.86	0.44	95.22
ResNet101	94.85 ± 1.89	94.96 ± 2.65	94.85 ± 2.68	94.84 ± 2.68	94.85 ± 2.68	0.60	93.85
SqueezeNet	93.89 ± 2.05	93.90 ± 2.90	93.89 ± 2.90	93.89 ± 2.90	93.89 ± 2.90	0.28	93.02

**Table 4:** Different trained CNN network parameters.

Network	Parameters (Millions)
InceptionV3	23.9
MobileNetV2	3.5
Densnet201	20
ResNet18	11.7
ResNet50	25.6
ResNet101	44.6
SqueezeNet	1.24

**Table 5:** Comparison of the findings of this study with those of other recent similar works.

Reference	Pre-Processing/Features	Classifier/Technique	Accuracy
Waranyu et al. [40] (2007)	red blood cell, a reticulocyte and platelet	ANN and genetic programming	87.22%
Damrongrit et al. [41] (2007)	HPLC	C4.5 decision tree, a naive Bayes classifier and MLP	93.23%
Fatemeh Yousefian et al. [42] (2010)	HbA2	MLP	87.40%
Paokanta et al. [43] (2011)	HbA2 and Genotype	MLP, KNN and naive Bayes	86.61%
Elshami et al. [10] (2012)	CBC	decision tree, naive Bayes and ANN	93.70%
Hosseini et al. [44] (2016)	CBC	MLP	92%
Marzuki et al. [45] (2017)	CBC and haemoglobin	active contour	90%
Borah et al. [46] (2018)	MCV	KNN and SVM	95.71%
Leila et al. [38] (2019)	CBC, MCH	ANN	92.50%
Reena et al. [39] (2020)	haematological parameters	decision tree, naive Bayes and ANN	91.74%
Proposed approach	Hb electrophoresis test	CNN (InceptionV3)	95.8%

## Data Availability

Not applicable.
